# The process of overcoming challenges faced by midwives providing perinatal mental health care in obstetric facilities in Okinawa, Japan: A qualitative study

**DOI:** 10.1111/jjns.70053

**Published:** 2026-04-30

**Authors:** Hisami Shimonaka, Matsuyo Inoue, Masaki Shinjo, Jun Kobayashi

**Affiliations:** ^1^ Graduate School of Health Sciences University of the Ryukyus Ginowan Japan; ^2^ Department of Nursing Okinawa Prefectural College of Nursing Naha Japan

**Keywords:** education, nursing, continuing, mental health, midwifery, perinatal care, qualitative research, reflective practice

## Abstract

**Aim:**

This study aimed to clarify how midwives overcome challenges in providing perinatal mental health (PMH) care in clinical settings and to inform feasible, context‐sensitive educational support.

**Methods:**

We used an interpretivist–constructivist qualitative design with a sequential two‐stage approach. Thirteen midwives working at five obstetric facilities in Okinawa, Japan, each with more than 2 years of PMH care experience, participated in one focus group discussion (FGD) and one in‐depth interview (IDI). FGD transcripts were analyzed descriptively to clarify key challenges and concerns and to inform the development of IDI questions. IDI transcripts were analyzed using reflexive thematic analysis to explore how midwives navigated and overcame these challenges, thereby generating an interpretive account aligned with the study aim.

**Results:**

Five themes were generated, from which we constructed a dynamic thematic map illustrating how challenges were overcome: contextual challenges triggered action; team‐based support, reflective practice, and feedback from clinical cases interacted recursively; and motivational substrates underpinned these processes and were reinforced through ongoing practice, sustaining midwives' involvement in PMH care under conditions of uncertainty. Reported barriers included limited opportunities for reflective dialogue within shift‐based workflows and limited visibility of post‐discharge outcomes.

**Conclusion:**

This study clarified the contextual challenges in PMH care and the key elements for overcoming them. These findings inform educational and organizational support combining flexible access to knowledge resources with interprofessional collaborative reflection grounded in psychological safety in emotionally and time‐constrained obstetric settings, enabling midwives to sustain engagement in PMH care under conditions of uncertainty.

## 1 INTRODUCTION

Perinatal mental health (PMH) care has become a global priority (World Health Organization [WHO, 2008]), and it has also become a national priority in Japan. Professional societies have issued guidelines and consensus statements and have expanded interprofessional training to strengthen PMH care (Japan Society of Obstetrics and Gynecology & Japan Association of Obstetricians and Gynecologists, 2017; Japanese Society of Perinatal Mental Health, 2017; Suzuki, 2024; Suzuki et al., 2018). At the policy level, the government promotes seamless, continuous support through coordinated, multiprofessional, cross‐sector pathways (Children and Families Agency, 2024). Yet empirical evidence remains limited regarding how Japanese midwives—key frontline providers—enact PMH care in day‐to‐day practice. Qualitative studies are lacking that examine midwives' firsthand experiences and the organizational and interprofessional contexts in which they work. Given reports that hospital‐based midwives in Japan often have limited time to provide care for women with uncomplicated pregnancies (Shiraishi, 2016), understanding what midwives experience in clinical settings is essential for deriving practical implications for feasible educational support that reflects real‐world practice.

PMH is critical because maternal psychological difficulties can affect both women and infants and, in severe cases, are associated with suicide risk (WHO, 2008). Countries such as the United Kingdom and Australia have developed advanced guidelines and service models (National Institute for Health and Care Excellence, 2014, Austin et al., 2017). Similar international efforts have expanded globally (Department of Health, 2016; Griffen et al., 2021; Høivik et al., 2021; Honikman et al., 2012; Rahman et al., 2013), informing PMH policy and practice across diverse health systems, including Japan. As these initiatives progress, various challenges—including those experienced by midwives as care providers—have been reported (Coates & Foureur, 2019; Viveiros & Darling, 2019).

Midwives face challenges throughout the PMH care pathway—from screening to ongoing support and referral—stemming not only from individual‐level factors (e.g., limited knowledge and skills) but also from system‐level barriers (e.g., time constraints, unclear referral pathways) (Bayrampour et al., 2018; Noonan et al., 2017). Many of these difficulties exceed what individual effort can resolve. The emotional labor of caring for women at risk of mental health problems can also contribute to burnout among midwives (Mollart et al., 2009; Mollart et al., 2013). At the same time, how midwives sustain their engagement in PMH care despite these burdens, including the motivational factors that support continued practice, remains insufficiently understood. Therefore, educational support aimed at preventing burnout and enhancing midwives' competencies is crucial. In line with this, several studies have reported that midwives themselves are seeking such support (Hauck et al., 2015; Noonan et al., 2018).

Although various initiatives have sought to enhance continuing education for midwives in PMH care (Alderdice et al., 2013; Shinohara et al., 2022; Wang et al., 2022; Yamashita et al., 2007), effective implementation strategies remain underdeveloped (Hauck et al., 2015; Legere et al., 2017). Despite widespread recognition of the need, time constraints continue to be a major barrier across continuing nursing education (Coventry et al., 2015; Shahhosseini & Hamzehgardeshi, 2014). Global shortages and uneven distribution of mental health professionals have been reported (WHO, 2018). In this context, securing protected time for midwives to engage in educational activities is difficult, and pragmatic, feasible training programs remain scarce.

To develop feasible educational strategies that address these challenges in real‐world clinical settings, it is essential to clarify how midwives overcome barriers in providing PMH care. Therefore, this study aims to clarify the processes by which midwives overcome challenges in providing PMH care and to inform the development of educational support programs by specifying key components of continuing education that can enhance PMH care competencies in clinical settings.

## 2 METHODS

### 2.1 Research design and methodology

We used a qualitative design with an interpretivist–constructivist stance. We used a sequential, complementary two‐stage design: focus group discussions (FGDs) mapped shared understandings and practice challenges that sensitized—without constraining—subsequent in‐depth interviews (IDIs), which supported deeper interpretations. IDIs were analyzed using reflexive thematic analysis (RTA), treating themes as analytically constructed and engaging researcher subjectivity through reflexive practice (Braun & Clarke, 2006, 2020, 2021). Rigor rested on reflexive dialogue, memoing, and an audit trail, rather than inter‐coder agreement or theme “validation” (Braun & Clarke, 2024).

### 2.2 Definitions and scope of PMH in this study

We adopt the WHO's broad conceptualization of PMH—promotion of well‐being and prevention, early identification, and appropriate response and care during pregnancy and up to 12 months postpartum (WHO, 2022). We focused on cases identified by participating midwives—via clinical judgment and local screening—as needing mental health support within routine care. Interviews explored challenges and how midwives overcame them. As we did not evaluate condition‐specific effects, the processes identified may not apply uniformly across PMH presentations.

### 2.3 Participants

Participants were midwives providing PMH care at obstetric facilities in Okinawa, Japan. Using purposive sampling, we sought information‐rich cases aligned with the study aim. Inclusion criteria were: (a) ≥2 years' PMH experience at their current facility; (b) prior participation in PMH‐related training; (c) capacity to join an FGD and an IDI. These criteria were intended to elicit reflective, practice‐based accounts of PMH care and perceived educational supports and gaps, grounded in sustained engagement in such work, where reflective meaning‐making had developed over time. We aimed to include variation in facility type, role, and years of experience; however, practical constraints limited the extent of diversity achieved.

The setting comprised primary and secondary obstetric facilities in Okinawa. Region selection involved convenience but was theoretically appropriate given local indicators of heightened perinatal need and limited specialist resources (Okinawa Prefecture, 2022, 2024). Tertiary facilities were not included, reflecting the study's focus on routine PMH care within obstetric settings managing primarily routine rather than complex cases.

Participants were recruited through nursing directors and unit managers at eight facilities. With institutional permission, managers were asked to suggest about three eligible midwives. Suggested staff then received information directly from the first author; participation was voluntary and enrolment followed written consent. Institutional consent was obtained from five facilities; 13 midwives consented.

### 2.4 Data collection

All participants completed one FGD and one IDI. IDIs were conducted within approximately 1 month of each participant's FGD. FGDs were online with three to five midwives per group (one per facility), three sessions total. IDIs were online or face to face per preference. Online interviews were video recorded; in‐person sessions were audio recorded. All data were transcribed and verified by participants.

Data were collected between October 2022 and March 2023. Demographic data (age, years of midwifery experience, tenure at the facility, position) were collected electronically and anonymized. All interviews were conducted by the first author, a midwife with clinical, educational, and PMH training‐development experience. FGDs examined PMH‐care challenges and resources (training, institutional systems); identified challenges informed the interview guide. IDIs then explored how midwives addressed these challenges using a semi‐structured format. For example, IDI prompts invited participants to reflect on challenges raised in the FGDs, explore whether they had encountered similar situations, and describe how they worked through those challenges, with follow‐up questions expanding on issues introduced by the participants themselves.

### 2.5 Analytic approaches

FGDs were analyzed descriptively to map challenge areas that informed the IDI guide and acted as sensitizing concepts (Sandelowski, 2000).

IDI transcripts were analyzed using RTA to identify patterns of meaning in how midwives overcame PMH‐care challenges (Braun & Clarke, 2006, 2021). Analysis proceeded iteratively at the semantic–latent interface through familiarization, memo‐supported initial coding, crafting candidate patterns, dataset‐wide review, and defining and naming themes (Braun & Clarke, 2021, 2024).

The first author coded the first five interviews case by case and mapped each trajectory of “working to overcome.” After each case, mapping and coding decisions were discussed in reflexive dialogue with a senior qualitative methodologist (JK). When interpretations diverged, alternative readings were explicitly compared, and theme boundaries and labels were refined through discussion until we developed a coherent account that best fitted the dataset. From these five cases, provisional cross‐case themes were constructed. The remaining eight interviews were analyzed recursively to test and refine provisional themes, consider rival explanations, and rename or redefine boundaries where warranted. FGD insights sensitized—without constraining—subsequent IDI coding; convergence and divergence between sources were examined so the developing model reflected the whole dataset. Decisions and products were retained as a multi‐component audit trail.

### 2.6 Reflexivity, data management, and adequacy

The first author maintained reflexive memos and engaged in regular reflexive dialogue with co‐authors and peers. For FGDs, NVivo (version 14.0, QSR International) supported coding and categorization to produce descriptive matrices. For IDIs, initial coding was undertaken in Excel; transcripts and codes were then imported into NVivo for further coding and theme development. We did not use automated text‐analytics functions. Participants verified their own transcripts, not final themes. As part of reflexive practice, three participants reviewed a draft of the manuscript and provided feedback on the interpretive themes; this input was used to support researcher reflection, not to validate findings. Consistent with RTA, we did not compute inter‐coder agreement or pursue theme “validation”; rigor rested on reflexive practice, coherence, and transparency (Braun & Clarke, 2024). We did not target saturation; analytic adequacy was judged when later interviews no longer reshaped core components relevant to the study aims (Braun & Clarke, 2024).

### 2.7 Ethical considerations

This study was approved by the Ethics Review Committee for Life Sciences and Medical Research Involving Human Subjects, University of the Ryukyus (Approval No. 1992). Participants received written and verbal information about the study purpose, procedures, potential burdens and compensation, data handling and publication, voluntary participation, and the right to withdraw. Written informed consent was obtained. Recordings were made only with consent. Participants were asked to keep focus‐group discussions confidential and could review, confirm, and revise their transcripts and indicate the extent to which their data would be included in publications.

## 3 RESULTS

### 3.1 Participant characteristics

The study participants were 13 midwives (mean age, 39.6 years; standard deviation [SD], 6.4; range, 30–47) (Table 1). The average duration of midwifery experience was 12.0 years (SD, 6.3; range, 3–22), and the average duration of employment at their current workplace was 11.3 years (SD, 5.1; range, 2–21).

**TABLE 1:** Participant attributes.

ID	Age (years)	Midwifery experience (years)	Years at current facility (years)	Position/ role on Ward	Interview duration (minutes)
A1	31	9	9	Manager	79
A2	31	9	2	None	81
B1	46	21	21	None	84
B2	34	9	9	None	80
B3	44	9	9	None	81
C1	43	17	13	Manager	59
C2	46	18	12	None	62
D1	44	20	20	Manager	83
D2	33	6	6	None	71
D3	47	22	16	Manager	79
E1	30	3	7	Education Coordinator	77
E2	41	6	11	None	81
E3	45	7	12	Manager	96

### 3.2 Challenges in providing PMH care

Based on data from FGDs, the challenges in providing PMH care were categorized into the following four areas: (1) Lack of mental health literacy, (2) Psychological burden on midwives providing PMH care, (3) Systemic challenges in providing PMH care within obstetric facilities, and (4) Difficulties in collaboration with community resources (Table 2).

**TABLE 2:** Challenges in providing PMH care.

Category	Subcategory
Lack of Mental Health Literacy	Limited PMH knowledge and skills
	Educational needs and requests for training
Psychological Burden on Midwives Providing Mental Health Care	Psychological burden midwives experience when providing care
	Concern about burden being concentrated on certain midwives
	Increased workload due to the large number of women needing support
Systemic Challenges in Providing PMH Care Within Obstetric Facilities	Limited capacity to provide care due to restricted time, space, and personnel
	Difficulties in coordination due to the increased number of team members, despite the need for multidisciplinary care within the facility
	Challenges in maintaining continuity of mental health care by the midwifery team
	Differences in midwives' competencies in providing mental health care
	Concerns about women who continue to be followed postpartum within obstetric facilities
	Worsening perinatal mental health and difficulty in providing support during the COVID‐19 pandemic
Difficulties in Collaboration with Community Services (Public Health Nurses)	Perceived mismatch in assessment between community and obstetric services
	Difficulty in handling situations when relationships between women and community supporters are strained
	Difficulty in sharing information with community services without consent from women
	Doubts and dissatisfaction about the community's response after sending support requests
	Increased difficulty in collaborating with community services due to the COVID‐19 pandemic

*Note*: Subcategories illustrate typical facets of each category; they are not exhaustive. Abbreviations: PMH, perinatal mental health.

### 3.3 Themes related to the process of overcoming challenges in PMH care practice

Five themes were generated from the IDI data (Table 3): (1) Background of challenges in PMH care, (2) Shared recognition of the ability to provide continuous care while supporting each other, (3) Contexts and practices that support ongoing reflective practice, (4) Feedback obtained from clinical cases, and (5) Motivational substrates sustaining PMH practice.

**TABLE 3:** The process by which midwives overcome challenges in providing PMH care.

	Theme		Category	
1.	Background of challenges in PMH care		Diverse backgrounds of women that affect PMH	
			Uncertainty in how to respond appropriately	
			Difficulties in engaging with perinatal women	
			Limitations of support within obstetric facilities	
			Emotional strain and burden experienced by midwives	
			Lack of opportunities to reflect on mental health care	
2.	Shared recognition of the ability to provide continuous care while supporting each other		Team‐based midwifery care with shared information on women requiring special support	
			Multidisciplinary collaboration for women with complex needs	
			Flexible team‐based care systems for improving perinatal support	
			Recognizing and adapting to one's facility's unique roles and resources	
			Coordination with external professionals such as public health nurses	
3.	Contexts and practices that support ongoing reflective practice			
		(Subtheme) 3a. Practice exposure as the engine of reflection 3b. Informational scaffolds for reflection 3c. Relational safety and supportive supervision 3d. Self‐care to sustain reflective capacity	Opportunities to engage in PMH care practice	3a [3b, 3c]
			Opportunities for self‐reflection on practice	3a [3b, 3c]
			Knowledge and technical support for PMH care practice	3b [3c]
			Availability of useful resources for improving practical skills	3b [—]
			Supportive Space for Discussing Difficulties and Concerns	3c [3a]
			Workplace environment that cares for the emotional well‐being of midwives	3c [3d]
			Strategies for maintaining one's own mental health	3d [3c]
			Practical insights gained through reflection	— ^a^
4.	Feedback obtained from clinical cases		Confidence in practice gained through feedback	
			Feedback that led to improvements in practice	
			Feedback contributing to midwives' sense of inadequacy	
			Difficulty in obtaining feedback as a challenge	
5.	Motivational substrates sustaining PMH practice		Recognition of midwifery's social responsibility in mental health care	
			Sense of fulfillment and passion as a care provider	
			Commitment to the profession of midwifery	
			Commitment to workplace and team relationships	
			Support from family	
			Work as a necessary part of life	

*Note*: Theme 3 comprises four subthemes (3a–3d) constructed analytically across categories to clarify its conceptual structure and to reflect their role in sustaining reflective practice. In the rightmost column, primary links appear first and secondary links appear in brackets; an em dash (—) indicates no direct correspondence.

Abbreviation: PMH, perinatal mental health.

^a^ This category is cross‐cutting and relevant to all subthemes in Theme 3.

**FIGURE 1:**
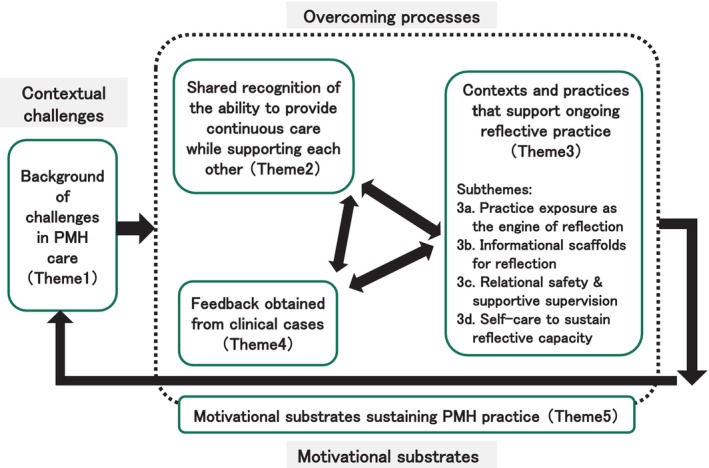
Dynamic thematic map illustrating the processes by which midwives overcame challenges in perinatal mental health (PMH) care. Contextual challenges (Theme 1) initiated action; shared recognition of the ability to provide continuous care while supporting each other (Theme 2), contexts and practices that support ongoing reflective practice (Theme 3), and feedback obtained from clinical cases. (Theme 4) interacted recursively rather than sequentially. Motivational substrates (Theme 5) underpinned these processes and could deepen through engagement. Arrows indicate typical, non‐deterministic linkages among themes through ongoing practice‐based interactions (e.g., handovers, brief huddles, case conferences, and supportive debriefings). Themes 1–5 are referred to as T1–T5 in the Results section.

To align our findings with the process‐oriented aim of this study, we developed a dynamic thematic map (Figure 1) that illustrates the temporal and structural relationships among the themes. This map serves as an interpretive aid consistent with RTA, and presents three higher‐order groupings:

Contextual challenges (T1): Background factors that trigger action, including diverse maternal needs and systemic limitations.

Overcoming processes (T2–T4): Team‐based support, reflective practice, and clinical feedback—presented in parallel to emphasize their interrelation and recursive nature.

Motivational substrates (T5): Foundational elements that pre‐exist and deepen through engagement, sustaining midwives' ongoing commitment to PMH care.

In this model, Contextual challenges (T1) initiate action; Overcoming processes (T2–T4) interact recursively rather than sequentially; and Motivational substrates (T5) provide foundational support that underpins T2–T4 and evolves through engagement in those processes. Arrows in Figure 1 indicate typical—not deterministic—linkages and recursive cycles. These linkages were enacted through ongoing practice‐based interactions.

Participants' accounts showed that team support, reflective practice, and clinical feedback interacted as midwives provided PMH care. One midwife described how sharing information about women of concern within the team enhanced staff members' awareness and observation, leading to changes in care approaches. Another explained how she incorporated practices and responses observed during external training into her own setting and interpreted women's and families' subsequent actions as feedback for refining her practice. For example, B1's description of briefly discussing cases during short breaks between shifts illustrates how everyday team consultation (T2) could simultaneously prompt reflection (T3), underscoring the recursive link indicated in Figure 1. In these ways, team support, reflective practice, and clinical feedback were experienced as interconnected, mutually shaping processes—rather than separate stages—within everyday PMH care.

To clarify the conceptual structure of Theme 3 (reflective practice), we generated four subthemes: (3a) Practice exposure as the engine of reflection, (3b) Informational scaffolds for reflection, (3c) Relational safety and supportive supervision, and (3d) Self‐care to sustain reflective capacity. These subthemes show how reflective practice develops and is maintained and how self‐care is fostered through collegial dialogue and supportive supervision.

The following sections provide detailed descriptions of each theme.

#### 3.3.1 Theme 1. Background of challenges in PMH care

This theme captures multifaceted background factors shaping PMH care challenges for midwives. Midwives recognized that the diverse biological, psychological, and social backgrounds of women influence their mental health in different ways, and this awareness formed a foundation for support. Participants also noted clear limitations within obstetric facilities in providing continuous support beyond pregnancy and childbirth. Amid these contexts, midwives often felt uncertain about how to provide appropriate care, experienced difficulties in adequately responding to women's needs, and carried both professional responsibility and emotional burden. In addition, opportunities for reflection were limited (Table 3).

Participants described factors influencing women's mental health, including prior psychiatric diagnoses, developmental traits, perinatal loss, adverse childhood experiences, intimate partner violence, and socioeconomic difficulties.

Based on these backgrounds, participants described three main challenges. First, because each woman's situation was unique, midwives often felt uncertain about what constituted appropriate care, leading to hesitation and anxiety. Second, they faced emotionally burdensome, hard‐to‐manage situations, such as caring for women with complex psychosocial backgrounds or being involved in births accompanied by perinatal loss. Third, although midwives recognized the need for continuous support, time, role, and system constraints within obstetric facilities made it difficult for them to remain sufficiently involved. These challenges contributed to emotional fatigue, loss of confidence, and feelings of helplessness. The following quote illustrates the psychological burden that arises from this uncertainty:

“When I talk with high‐risk women, I often worry about how even a single word I say might be interpreted. … Not knowing what the right answer is makes me keep thinking—and maybe that's part of what makes it feel so emotionally heavy.” (E3)

Participants also reported very limited opportunities to reflect on their own mental health care practices. The following quote illustrates the lack of space for reflection in shift‐based work settings, where care was handed over among staff:

“Even when things turn out well or go in a slightly different direction, I often don't know whether it was because of what I did or the result of everyone's combined efforts. It just ends without any clarity. … Looking back, I feel like I've rarely had the chance to reflect on how my own actions actually played out.” (D1)

This theme highlights personal and contextual influences on women's mental health and shows how these factors generate diverse, care‐shaping challenges for midwives in PMH practice. Strategies to address these challenges appear in the following themes.

#### 3.3.2 Theme 2. Shared recognition of the ability to provide continuous care while supporting each other

This theme highlights the shared recognition among midwives and other professionals—within and beyond facilities—that they can provide continuous care through mutual support throughout the care process (Table 3).

This recognition transcended differences in staffing models, professional roles, and care systems. Facilities varied in whether prenatal care was provided by a consistent midwife or rotating staff, and in the availability of psychologists and social workers. In some settings these professionals were absent or not dedicated to maternity care, leaving midwives to fill the gaps. Still, participants emphasized the importance of “seeing the woman together” as a team.

Midwives shared information about women requiring special support during handovers and meetings, enabling multi‐perspective care and a sense of reassurance among team members through team consultation:

“We're now at a point where we can all observe the women together. It's not exactly about overcoming something, but having that sense of ‘we're all watching together’ helps both us and the mothers… They're seen from different perspectives—not just by one person, but by all of us.” (A1)

In complex cases, midwives collaborated with doctors, clinical psychologists, and other professionals to determine care plans. Team‐based decision‐making helped reduce the psychological burden on individual midwives:

“For difficult cases, we hold conferences and consult with doctors to decide on care plans… so, I'm not carrying it all by myself. In that sense, I think it really helps.” (B1)

In facilities without social workers, participants assumed social work responsibilities and reframed this expanded role as a strength.

Collaboration with external professionals, such as public health nurses, provided reassurance that women would continue to receive care after discharge:

“Because the public health nurses are there, we feel reassured that the women will be okay after leaving the hospital… we can send them off with peace of mind.” (E2)

Overall, this theme underscores how shared recognition and mutual support—regardless of institutional differences—play a vital role in overcoming challenges in PMH care.

#### 3.3.3 Theme 3. Contexts and practices that support ongoing reflective practice

This theme focuses on how midwives enhance clinical competencies through ongoing reflection in challenging settings. Reflective practice was not an isolated cognitive activity but a socially and structurally supported process (Table 3). Practice exposure, informational resources, relational safety, and self‐care interacted dynamically to sustain reflective capacity. Practical insights—such as improved communication strategies—were generated through these interconnected elements, reinforcing iterative learning and care. To illustrate this pattern, the findings are organized into four subthemes.

##### Subtheme 3a. Practice exposure as the engine of reflection

PMH care encounters (e.g., interviews, consultations) triggered reflection and initiated experiential learning. Advice from senior midwives and hands‐on experiences helped participants identify gaps and improve care:

“We often talk to each other, even during short breaks between shifts. Like, ‘There's a patient like this,’ and so on… I think we are able to talk among staff members.” (B1)

“At first, I probably was not able to identify what was a problem for the patient. But then, after being told by my senior, I started to realize, ‘Oh, I should have asked about that too,’ and I think that is how I reflected on it.” (A2)

##### Subtheme 3b. Informational scaffolds for reflection

Access to knowledge and resources strengthened reflection. Midwives learned from colleagues' records, observed others' practices, exchanged ideas during training, and sought external information:

“I read patient narratives and checked online about grief care—what other hospitals do—and thought about what we could do here.” (A1)

“I used to sit behind senior midwives during consultations, just listening and thinking, ‘Oh, this is how they do it.’ That was in my first couple of years.” (B1)

##### Subtheme 3c. Relational safety and supportive supervision

Psychological safety and approachable colleagues enabled midwives to share doubts and seek advice. Recognition and emotional support from supervisors and peers were crucial:

“It really made a difference when staff or the head nurse said, ‘You did well,’ or ‘Thank you for your work.’ If they had not said anything, it might have been really tough.” (C1)

“Every time someone said, ‘It is not your fault,’ I cried my eyes out. The seniors would listen to me and tell me, ‘Yes, that happens sometimes,’ and they always listened to me, and somehow, I got through it.” (D2)

##### Subtheme 3d. Self‐care to sustain reflective capacity

Midwives used personal strategies to manage emotional strain and maintain mental space for reflection. Talking through difficult cases and setting boundaries helped prevent burnout:

“There are heavy cases—… But by talking it out in detail with co‐workers, and then with supervisors too, I can process it. I do not carry that weight home.” (D2)

Another participant described learning to “switch” attention away from rumination after a preceptor normalized similar experiences.

This theme illustrates how midwives overcome practical challenges through sustained reflective practice—processing difficulties, learning from experience, and applying insights to future care. The continuity of reflection was supported by a dynamic interplay of practice exposure, informational resources, relational safety, and self‐care.

#### 3.3.4 Theme 4. Feedback obtained from clinical cases

This theme explores how feedback from clinical cases triggered reflection, learning, and practice adjustments. Feedback from mothers, families, collaborating professionals, and related institutions supported reflective practice, enhanced confidence, and improved care quality. At the same time, the study revealed challenges in obtaining feedback and the emotional burden associated with its absence or negativity (Table 3).

Some participants described how subtle or indirect feedback affirmed the value of their care and led them to recognize their supportive role. One participant reflected on a moment during an abortion procedure:

“I heard indirectly that she said it was good that I was there during the procedure. I might have just been sitting next to her, but I felt that even that could be a small source of support for mothers.” (A1)

Another participant shared how seeing a mother return for a follow‐up visit reassured her that her care had been appropriate:

“Sometimes I worry—like, will she be okay until next time? I might send her home feeling anxious. But when I see that she came back safely, I feel relieved. If there's no major incident or mistake, maybe this level of care is okay.” (D1)

Participants who were continuously involved with mothers from pregnancy to postpartum described how sustained engagement allowed them to build trust and understand the mothers more deeply:

“Being able to stay involved continuously is really significant. Even if things don't go well this time, there's always a next time. So, by waiting patiently, maybe we can build a connection.” (B1)

In some cases, emotionally difficult feedback prompted participants to reflect and improve their practice. One participant described how a mother's strong reaction led her to adjust her approach:

“When I talked to a patient about …, she got really angry. After that, she stopped listening completely. I was probably the trigger, and afterward she refused care from the staff. …Since then, I've become much more careful about how I respond and consider the background of each patient.” (D2)

Feedback also included emotionally complex situations. One participant described conflict and sadness when supporting a mother who said she would not raise her baby and later faced the same situation again:

“Last time, we tried various interventions like saying, ‘Let's try not to have another unintended pregnancy,’ but it ended up the same. We're involved now, but it feels like it'll be just like last time. It's kind of sad—this sense that what we're doing won't bear fruit.” (E3)

Participants also noted the difficulty of obtaining feedback, especially after discharge. Not knowing how mothers were doing in the community created a sense of emotional uncertainty:

“After we refer women to the public health nurse, we often have limited information about what happens afterward in the community. It's a shared concern among us.” (A2)

Feedback also emerged after adapting practices based on reflection and external training. One participant described introducing bereavement care elements observed during training and interpreting follow‐up gestures from women and families as affirming feedback.

Overall, participants described that feedback—whether positive, negative, or absent—informed their evaluation of care, ongoing learning, and emotional wellbeing.

#### 3.3.5 Theme 5. Motivational substrates sustaining PMH practice

This theme focuses on the diverse and deeply personal motivations that drive participants to continue providing PMH care. Despite challenges, participants found meaning through social responsibility, fulfillment from support, professional commitment, and workplace/family support (Table 3). These motivations, rooted in individual contexts, sustained ongoing practice and efforts to overcome difficulties.

The participants described a wide range of reasons for continuing their practice despite challenges. They recognized the social role of midwives in PMH care and felt a sense of mission. Purpose and reward from supporting women's mental health motivated continued engagement. In addition, commitment to midwifery, attachment to the workplace, family support, and practical livelihood needs also contributed. These elements varied by individual, forming the basis for continued involvement despite difficulties.

One participant described the social role of midwives:

“Midwives study specialized knowledge to care for mothers, pregnant women, and babies from pregnancy through the postpartum period. So, I think this is just what we are supposed to do… I guess the expectation is that as a midwife, we are required to be able to handle this kind of mental health care.” (C2)

Another participant reflected on her strong emotional attachment to the profession:

“I guess I really love this job. Even when I face emotionally heavy situations, I just think, ‘Well, this is part of being a midwife,’ and I accept it. So, I do not feel like quitting or giving up.” (E3)

A different participant spoke of personal motivations rooted in both practical and relational factors:

“Honestly, the biggest thing is that if I quit, I would not be able to make a living… But maybe it is also that I have someone at work I can really say whatever I want to, not just complaints, but honestly say what I feel. That might be a big part of it.” (D3)

These motivations were often described alongside workplace relationships and opportunities to talk through difficulties.

This theme highlights the diverse motivations to sustain midwives' commitment to PMH care, even amid the challenges in Theme 1. These motivations closely relate to team‐based support (Theme 2), reflective practice (Theme 3), and clinical feedback (Theme 4), which underpin sustained PMH practice in clinical settings.

## 4 DISCUSSION

### 4.1 Theoretical interpretation of the themes (overview)

This section offers a concise, process‐oriented interpretation of how midwives navigate and remain engaged in PMH care, a field characterized by persistent uncertainty and the absence of a single “right answer.” Across Themes 1–5, the findings indicate practice conducted under conditions of limited opportunities for reflection and complex clinical contexts. Responses to these conditions unfolded through recursive interactions among team‐based support, reflective practices, clinical feedback, and motivational substrates that both preconditioned and emerged through ongoing participation in PMH care. Together, the themes delineate a process through which midwives made sense of challenges, iteratively adapted their care, and maintained engagement in PMH despite enduring institutional constraints.

#### 4.1.1 Theme 1: Sustained uncertainty under constrained reflection

The challenges identified in the FGDs—including limited mental health literacy, psychological burden, systemic constraints within obstetric facilities, and difficulties in collaboration with community resources—are consistent with prior research on midwives providing PMH care (e.g., Bayrampour et al., 2018; Noonan et al., 2017), confirming the substantial professional and emotional demands involved.

Building on these descriptive findings, Theme 1 offers a deeper interpretive understanding of how such challenges are experienced and sustained in practice. The in‐depth interviews revealed that the diversity and complexity of women's biological, psychological, and social backgrounds required highly individualized responses, making it difficult for midwives to determine what constituted appropriate care in each situation. This context generated a pervasive sense of situational uncertainty, as midwives perceived that there were no clear or standardized “right answers” in PMH care.

A central contribution of this study lies in its conceptualization of this “background of challenges” as initial conditions that structure midwives' ongoing sense‐making, help‐seeking behaviors, and interpretive judgment throughout PMH care. The data further indicate that limited opportunities for reflective dialogue and timely feedback within shift‐based workflows may have impeded the resolution of such uncertainty. As a result, unresolved uncertainty could accumulate as an affective–cognitive load, potentially influencing subsequent collaborative engagement, learning from clinical cases, and the degree to which midwives were able—or willing—to sustain their involvement in PMH care despite persistent ambiguity.

#### 4.1.2 Themes 2–4: Recursive interactions among team‐based support, reflective practice, and feedback

Themes 2–4 elucidate recursive and mutually reinforcing interactions among team‐based support, contexts and practices that enable ongoing reflective engagement, and multi‐source feedback derived from clinical cases. Rather than constituting a linear sequence, these processes were enacted through routine clinical encounters—such as handovers, brief huddles during shift transitions, case conferences, and supportive debriefings—in which midwives externalized uncertainty, shared observations, processed emotional responses, and engaged in the joint interpretation of evolving clinical situations (Figure 1). Through such interactions, ambiguity was transformed into provisional yet actionable insights, while emotional load was distributed across members of the care team.

These dynamics align with Edmondson's (2012) conceptualization of teaming and psychological safety, which underscores the relational conditions that enable speaking up, help‐seeking, and collective learning within fluid and interdependent work environments. Psychologically safe forums and supportive relationships with colleagues and collaborating professionals emerged as essential conditions for addressing uncertainty and maintaining engagement in PMH care. At the same time, the findings resonate with experiential and reflective learning theories (Kolb, 1984; Schön, 1983/2007), which conceptualize learning as an iterative cycle through which experience is reflected upon, interpreted, and subsequently adapted. Within this framework, informational scaffolds—including access to resources, documentation by senior colleagues, observation of expert practice, feedback, debriefings, and external training—served to anchor structured reflection and support autonomous clinical reasoning under real‐world constraints.

Importantly, feedback in PMH care extended beyond explicit evaluations from women, encompassing the evolving responses of women and families and the assessments of collaborating professionals and institutions. Through the shared interpretation of these diverse feedback sources, midwives consolidated clinical confidence in some cases and revised or recalibrated their approaches in others, thereby maintaining ongoing cycles of learning and adaptive practice. However, the data also reveal clear boundary conditions. When brief but regular forums for dialogue were available and psychological safety was maintained, uncertainty could be jointly interpreted and transformed into learning. In contrast, when time for reflection was limited and post‐discharge feedback remained opaque, uncertainty tended to persist, amplifying emotional strain and constraining reflective learning processes.

#### 4.1.3 Theme 5: Motivational substrates as conditions and emergent outcomes

Theme 5 highlights the multi‐layered motivational substrates that supported midwives' sustained engagement in PMH care, including a sense of professional mission, attachment to the midwifery role, supportive workplace relationships, family support, and, for some, practical considerations such as financial necessity. These findings are consistent with prior research emphasizing professional identity and meaning in work as stabilizing resources in emotionally demanding contexts (Hunter, 2004; Hunter & Warren, 2014). Extending this literature, motivational substrates are conceptualized here as a dynamic substrate—a resource that both underpins and is reshaped through participation in practice.

Participants' narratives indicated that motivational resources underpinned their willingness to engage in team dialogue, maintain reflective practice, and learn through feedback. At the same time, supportive or affirming experiences—such as collegial encouragement, constructive feedback, or recognition of one's contribution to women's coping—served to reinforce and deepen motivation. Thus, motivation functioned simultaneously as a precondition enabling engagement and as an emergent outcome of ongoing practice, helping midwives remain involved in PMH care despite persistent uncertainty and contextual constraints.

### 4.2 Implications for educational support grounded in the qualitative findings

#### 4.2.1 Supporting reflective learning embedded in everyday clinical practice

Grounded in the present findings, educational support for PMH care should be designed to fit midwives' everyday clinical contexts, where time constraints, emotional demands, and uncertainty are pervasive. Rather than relying solely on discrete training events, educational support is more sustainable when embedded within ongoing experiential learning and connected to routine clinical practice.

Because participants described learning and reflection as occurring through brief, informal interactions embedded in daily work, the educational directions suggested here do not necessarily require additional protected time or large‐scale programs. Instead, flexible and asynchronous learning resources may enhance feasibility by complementing existing reflective practices rather than adding separate educational demands.

The findings suggest that knowledge resources can function as informational scaffolding to support reflective practice under cognitive load. In contexts where time for reflection is limited (Theme 1), educational strategies that enable flexible access to essential information can help midwives prepare for difficult encounters and structure reflection afterward. Such strategies include short, asynchronous learning resources integrated into existing continuing professional development routines; their effectiveness warrants examination in future implementation studies.

#### 4.2.2 Linking individual learning to collaborative reflection and feedback through psychological safety

The findings further indicate that educational support should explicitly link individual learning to collaborative reflection in practice. Midwives described learning through dialogue in handovers, brief conversations, and case discussions (Themes 2–3), suggesting that reflection was enacted as a shared, relational process rather than an individual cognitive activity.

Educational approaches that strengthen these reflective micro‐practices—such as brief, protected moments for team reflection, structured debriefing routines after emotionally challenging cases, or supervisor‐facilitated reflection—may help transform uncertainty into shared sense‐making. Importantly, these strategies require psychological safety to be workable (Edmondson, 2012). Leadership behaviors that may foster psychological safety in obstetric settings include openly acknowledging uncertainty, responding to concerns or mistakes as opportunities for learning rather than blame, and routinely recognizing staff efforts in emotionally demanding care.

The findings also highlight the practical importance of feedback systems. Since lack of feedback after discharge contributed to emotional uncertainty (Theme 4), educational support should be accompanied by organizational mechanisms that enable midwives to access meaningful feedback without increasing burden. Potential options include clearer pathways for information‐sharing with community services, brief templates for communicating key concerns, or opportunities to reflect on follow‐up outcomes when available. These approaches require collaborative development and evaluation in future work.

#### 4.2.3 Addressing emotional burden through interprofessional learning and trauma‐informed perspectives

Finally, the findings indicate that educational support for PMH care should account for both the emotional demands of practice and the boundary‐crossing nature of care delivery. Participants described emotionally demanding encounters and learning through observation, adaptation, and shared reflection, underscoring the importance of trauma‐informed and bereavement‐sensitive perspectives (Law et al., 2021).

Educational support should therefore attend not only to technical knowledge but also to emotional processing, self‐care, and provider well‐being that enable reflective capacity over time. In addition, because PMH care extends across facility and community boundaries, interprofessional learning should be scoped in ways that reflect how care is enacted in practice. Learning opportunities may involve midwives, obstetricians, public health nurses, psychologists, and social workers where available, with flexible formats aligned with existing referral pathways and postpartum follow‐up processes.

The findings suggest that such capacities are most effectively developed through supportive supervision and collegial dialogue rather than in isolation, aligning with prior work on learning through talk in workplace‐based professional development (Eppich et al., 2016).

### 4.3 Scholarly and practical contributions

This study elucidated how midwives maintain engagement in PMH care under conditions of uncertainty and highlighted support structures that connect practice, reflection, and collaboration. These findings extend prior research beyond individual‐level educational effects to encompass team‐based learning, reflective practices, and motivational substrates sustaining PMH practice in everyday clinical work.

Regional context may also have shaped these dynamics. In Okinawa, an island setting with relatively compact professional communities, close interprofessional and community‐based networks may facilitate informal consultation, rapid information sharing, and continuity of feedback beyond discharge, thereby supporting team‐based reflection and collaboration.

In Japan, policy efforts increasingly emphasize early identification of PMH needs and seamless multidisciplinary support to prevent maternal isolation, alongside expanded educational support for providers. While prior studies report short‐term educational effects (Shinohara et al., 2022), our findings suggest that learning is embedded in ongoing collaborative sense‐making within routine practice and sustained by relational and emotional conditions, including psychological safety and provider well‐being.

Taken together, this study adds a complementary perspective by highlighting that sustaining PMH support systems requires attention not only to women's needs but also to providers' well‐being through reflective and supportive team environments. Our findings also underscore the importance of further integrating trauma‐informed perspectives into perinatal support systems.

### 4.4 Implications for clinical nursing practice

The findings suggest several implications for clinical nursing practice in PMH care. First, multidisciplinary teamwork, rather than reliance on individual effort, appears essential for addressing complex PMH needs. Accordingly, organizational systems that facilitate teamwork may support more sustainable practice.

Second, reflective cultures supported by psychological safety underpin continuous professional growth in emotionally demanding contexts. Third, structured opportunities for obtaining and using feedback from women and clinical teams may contribute to improvements in care quality. Together, these conditions foster practice environments in which midwives can engage in PMH care with greater autonomy and confidence.

### 4.5 Limitations and future directions

This study has several limitations that should be considered when interpreting the findings. Participants were recruited from primary and secondary obstetric facilities within a single prefecture in Japan, and midwives with limited PMH experience or those working in tertiary‐level settings were not included. As PMH services, case complexity, and organizational contexts vary across regions and levels of care, the transferability of the findings should be considered in relation to contextual similarity rather than broad generalizability. In addition, the purposive inclusion of midwives with prior PMH training and at least 2 years of experience may have limited perspectives from early‐career midwives or those who disengaged from PMH care. Because participants were nominated by unit managers, the possibility of selection bias toward more motivated or experienced individuals cannot be excluded.

This study provides qualitative insights into processes through which midwives navigate uncertainty and sustain engagement in PMH care. Building on these findings, future research should examine the implementation and outcomes of educational support across diverse clinical settings and over time, including organizational conditions that enable reflective, collaborative, and psychologically safe PMH practice.

## 5 CONCLUSION

This study clarified the contextual challenges in PMH care and identified key elements through which midwives overcome them: team‐based support, reflective practices, feedback from clinical practice, and motivational substrates. These findings illuminate processes that enable midwives to sustain engagement in PMH care under conditions of uncertainty.

The results inform the development of educational and organizational support that combines flexible access to knowledge resources with interprofessional collaborative reflection grounded in psychological safety within emotionally and time‐constrained obstetric settings. By specifying core components relevant to continuing professional development, this study contributes to the advancement of continuing nursing education and offers practical directions for strengthening PMH care competencies in real‐world clinical contexts.

## AUTHOR CONTRIBUTIONS

Hisami Shimonaka led the study design, collected and analyzed the data, interpreted the findings, and drafted the manuscript. Matsuyo Inoue contributed to the design, interpretation, and writing. Masaki Shinjo contributed to the design, participated in discussions on applying the findings to educational programs, and assisted with writing. Jun Kobayashi supervised the study design, thematic analysis, interpretation including educational implications, and manuscript preparation. All authors approved the final manuscript.

## CONFLICT OF INTEREST STATEMENT

The authors declare no conflicts of interest in association with the present study.

## Data Availability

The datasets generated and analyzed during the current study are not publicly available due to ethical and privacy considerations, as the qualitative interview data contain potentially identifiable information.
